# Comprehensive fecal metabolomics and gut microbiota study of the protective mechanism of herbal pair *Polygonum hydropiper-Coptis chinensis* in rats with stress-induced gastric mucosal damage

**DOI:** 10.3389/fphar.2024.1435166

**Published:** 2024-08-13

**Authors:** Shouzhong Ren, Chenhui Ren, Yamei Zhao, Haiyan Niu, Yiqiang Xie

**Affiliations:** ^1^ Engineering Research Center of Tropical Medicine Innovation and Transformation of Ministry of Education, International Joint Research Center of Human-machine Intelligent Collaborative for Tumor Precision Diagnosis and Treatment of Hainan Province, Hainan Provincial Key Laboratory of Research and Development on Tropical Herbs, School of Pharmacy, Hainan Medical University, Haikou, Hainan, China; ^2^ School of Life Sciences, Hainan University, Haikou, Hainan, China; ^3^ Department of Pathology, The First Affiliated Hospital of Hainan Medical University, Haikou, Hainan, China; ^4^ College of Traditional Chinese Medicine, Hainan Medical University, Haikou, Hainan, China

**Keywords:** traditional Chinese medicine, gut microbiota, metabolomics, gastric mucosal lesion, gastroprotection

## Abstract

**Introduction:** Stress-related gastric mucosal lesions (SGMLs) are the most common complication in critical care patients. Previous studies have demonstrated that herbal pair (HP), *Polygonum hydropiper-Coptis chinensis* (HP P-C) has the anti-SGML effect. However, the underlying mechanism of HP P-C against SGML remains elusive. This study aimed to elucidate how HP P-C extracts exert their protective effects on SGML by examining the role of gut microbiota and metabolites.

**Methods: **SD rats were pretreated with different doses of HP P-C extracts for 6 days, followed by inducing SGML with water-immersion restraint stress (WIRS). After a comprehensive evaluation of serum and gastric tissue indicators in rats, 16S rRNA sequencing and metabolomics analyses were conducted to assess the impact of HP P-C on the fecal microorganisms and metabolites and their correlation.

**Results: **Animal experiment suggested that pretreatment with HP P-C effectively reduced the gastric mucosal lesions, remarkably increased superoxide dismutase (SOD) activity in SGML model rats induced by WIRS. 16S rRNA sequencing analysis showed that HP P-C altered the composition of gut microbiota by raising the abundance of *Lactobacillus* and *Akkermansia*. In addition, metabolomics data identified seventeen main differential metabolites related to WIRS-induced gastric mucosal injury, primarily involving in tyrosine metabolism and betalain biosynthesis. HP P-C was found to regulate tyrosine metabolism and betalain biosynthesis by down-regulating the tyramine, L-tyrosine and L-dopa and up -regulating the gentisic acid and dopaquinone.

**Conclusion:** Taken together, this study indicated that HP P-C could effectively protect against WIRS-induced gastric mucosal lesions by modulating intestinal flora and metabolites.

## 1 Introduction

Stress-related gastric mucosal damage is the most frequent complication in critical care patients, often leading to severe upper gastrointestinal bleeding or gastrointestinal perforation (Alhazzani et al., 2017) and is related to increased morbidity and mortality. Clinical data indicate that stress ulcers occur in 15%–50% of critical patients, typically triggered by strong events, including severe trauma, burns, shock, infection, exhaustive exercise, surgery, or severe dysfunction of internal organs. This result in erosion, bleeding, or superficial ulcers in gastric mucosa. Therefore, there is a growing interest in natural agents that could potentially prevent stress-related mucosal lesion. Traditional Chinese medicine (TCM) has obtained global recognition in recent years and is widely perceived as natural and safe with minimal side effects. Moreover, TCM has the advantage of multi-level and multi-target therapy, which may help avoid adverse reactions commonly associated with single-target therapies in Western medicine (Zhang J. et al., 2020).

In China, TCMs are commonly used to prevent and treat diseases related to gastric mucosal injury, including gastric ulcers and gastritis. *Polygonum hydropiper* Linn and *Coptis chinensis* Franch have been applied to treat gastrointestinal disorders for thousands of years and have demonstrated clinical efficacy with minimal adverse reactions. In modern Chinese clinical practice, *C. chinensis* is frequently employed for gastritis (Song et al., 2023), enteritis and gastric ulcers (Li et al., 2022). Modern research shows that *P. hydropiper* has a variety of pharmacological activities such as anti-inflammatory, antioxidant and gastric protective effect (Nasir et al., 2021; Kong, et al., 2023). Flavonoids are the primary bioactive substances in *P. hydropiper*, while alkaloids are the predominant active components of *C. chinensis* (Hao et al., 2020). It has been reported that these compounds possess antioxidant and anti-inflammatory properties, which are crucial for preventing gastric mucosal damage (Zhang et al., 2022). The previous studies have illustrated that *P. hydropiper* extract can mitigate alcohol-induced Ges-1 cell damage and gastric injury in rats (Ren et al., 2021). However, the detailed mechanism underlying the compatibility of the herbal pair, *P. hydropiper*-*C. chinensis* in treating gastric mucosal injury remains poorly understood. The exploration into the impact and mechanism of this herbal pair against gastric mucosal damage holds meaningful implications for advancing medical practice.

In the late years, integrated studies of metabolomics and the intestinal microorganisms have been extensively applied to illuminate the mechanisms of TCM (Feng et al., 2022; Yan et al., 2023). The metabolomics is important for tracing the biomarkers and identifying regulatory pathways linked to diseases. By analyzing and verifying specific disease biomarkers, it is possible to better understand the metabolic pathways of certain substances and elucidate their mechanisms of action (Xu et al., 2024). The intestinal microorganisms have the ability to influence gastrointestinal homeostasis, the immune system of hosts and the development of diseases. TCM interventions can modulate the community microbial flora structure and metabolism, reaching therapeutic benefits (Fan et al., 2020; Zhang W. et al., 2020).

The aim of the current study was to obtain comprehensive metabolic and gut microbiota profiles to thoroughly analyze the mechanism of HP P-C, an effective treatment for stress-induced gastric mucosal damage. For this purpose, water-immersion restraint stress (WIRS) models were utilized to assess the protective action of HP P-C on gastric mucosal lesion in rats. Then, by 16S rRNA sequencing and UPLC-MS/MS technology, the phenotype of the microflora and metabolome in WIRS rats was characterized to explore underlying mechanisms of the protective effects of HP P-C on gastric mucosal injury.

## 2 Materials and methods

### 2.1 Plant materials, extraction preparation and phytochemical analysis

The stems and leaves of *P. hydropiper* L (voucher number: 20190723) were collected in July 2019 in Sanya City, Hainan province of China. *Coptis chinensis* F (voucher number: 20190918, local: Sichuan, China) were purchased from Hainan Shounanshan Industry Co., Ltd. (Haikou, China) in September 2019. The two plants were authenticated by Prof. Niankai Zeng from Hainan Medical University. A voucher specimen was deposited the Laboratory of Traditional Chinese Medicine of Hainan Medical University. Dried plant material of HP P-C (mixture of 300 g *P. hydropiper* and 150 g *C. chinensis)* were refluxed with 60% ethanol for 1 h. The residue was then extracted twice under the same conditions. The ethanol extracts were combined and concentrated under reduced pressure. Then, the extract was purified using AB-8 macroporous resin with 60% ethanol. The ethanol elution fraction collected, dried and kept at −20°C for further analysis.

High performance liquid chromatography (HPLC) analysis was conducted on a Waters 2,690/5 System. Kromasil 100-5-C18 was used as the stationary phase. Mobile phase A was acetonitrile, and mobile phase B was 30 mmol/L ammonium bicarbonate with 0.7% ammonia water and 0.1% triethylamine (v/v). The elution procedure was set as follows: 0–40 min, 0% B-35% B; 40–50 min, 35% B; 50–51 min, 35% B-10% B; 51–55 min, 10% B. The flow speed was maintained at 1.0 mL/min. The injection volume was 20 µL. The detection wavelength was 345 nm. The unknown compounds are identified by comparing them with known standards.

### 2.2 Animals and experimental design

A total of 60 SD rats (180–220 g, male) were obtained from Changsha Tianqin Biotech Co., Ltd. (Changsha, China). The rats were kept in cages at 25°C with a 12-h alternating light/dark cycle and allowed *ad libitum* access to water and food. All animal experiments were conducted in accordance with the national guidelines for the care and use of laboratory animals and were approved by the Animal Ethics Committee of Hainan Medical University (No. HYLL-2022–365, Haikou, China).

Rats were randomly divided into six groups: the normal control group (NC), WIRS-model group (WIRS), ranitidine (30 mg/kg, Ran) and HP P-C low-dose (10 mg/kg, HP P-C-L), middle-dose (20 mg/kg, HP P-C-M), high-dose (40 mg/kg, HP P-C-H) groups. The animals were respectively administered HP P-C for 6 days at doses of 10, 20, 40 mg/kg body weight in the HP P-C groups, while the rats in the NC and WIRS groups were orally gave an equivalent volume (10 mL/kg) of distilled water. The WIRS model was established as follows. The rats were fasted for 24 h after 6 days of gavage. On the seventh day each rat in the groups except those in the NC group was restrained individually in a homemade cage with its head up. Then, the cage was vertically immersed in a constant temperature water bath (19°C ± 1°C) for 6 h, and the water level reached position of the rat’s xiphoid. After 6 h, the rats were euthanized under anesthesia with chloral hydrate (300 mg/kg, ip), and blood and stomach tissue samples were collected from the rats for biochemical and morphological analysis. The fecal samples of rats were collected and stored at −80°C for metabolite and gut flora analysis.

### 2.3 Assessment of the gastric lesion index

The stomach was removed rapidly, washed with saline and fixed on a platform for macroscopic observation. The total area (mm) of gastric mucosal injury was measured, and the degree of gastric mucosal lesion was represented as the gastric lesion index (GLI).

### 2.4 Histopathological examination

After the measurement of GLI, stomach tissue was fixed in 4% buffered paraformaldehyde, embedded in paraffin, cut to a thickness of 3 µm and stained with hematoxylin-eosin for histological observation under an optical microscope.

### 2.5 Assay of SOD activity

SOD activity in the serum were assessed by commercial assay kits according to the manufacturer’s protocol (Nanjing Jiancheng Bioengin Institute, Nanjing, China).

### 2.6 Metabolomics analysis

The fecal samples were prepared by mixing 50 mg of feces with 400 µL of methanol and ice-cold water (4:1), followed by vortexing and centrifugation at 13,000 rpm at 4°C for 15 min. The resulting supernatant was collected for UHPLC-Q Exactive HF-X/MS analysis. Quality control (QC) samples were employed to ensure the stability of sequence analysis. Chromatographic separation was achieved using a Waters ACQUITY HSS T3 (2.1 mm × 100 mm, 1.8 µm particle) analytical column at 40°C. The mobile phase consisted of 0.1% formic acid (v/v) in water: acetonitrile (95:5, v/v) (A) and 0.1% formic acid in acetonitrile: isopropanol: water (47.5:47.5, v/v) (B) at a flow rate of 0.40 mL⋅min^−1^. The injection volume was 3 μL. The mass spectrometric data were collected using a Thermo UHPLC-Q Exactive HF-X Mass Spectrometer equipped with an electrospray ionization (ESI) source. ESI was used in both positive and negative ion modes with capillary voltages of 3.5 kV and 3.5 kV, respectively. Data acquisition was performed with the Data Dependent Acquisition (DDA) mode.

The Liquid Chromatography-Mass Spectrometry (LC-MS) raw data was pre-processed by Progenesis QI (Waters, Milford, United States) software. The fecal metabolites were identified by searching the Human Metabolome Database (HMDB) (http://www.hmdb.ca/), Metlin and Majorbio databases. The data matrix obtained by searching database was uploaded to the Majorbio cloud platform (https://cloud.majorbio.com) for data analysis. The R package “ropls” (Version 1.6.2) was used to perform principal component analysis (PCA) and partial least squares discriminant analysis (PLS-DA), and 7-cycle interactive validation evaluating the stability of the model. The variables with a variable importance in projection (VIP) value > 1 and *p* < 0.05 from the PLS-DA analysis were considered as potential biomarkers of differential metabolites. The Kyoto Encyclopedia of Genes and Genomes database (KEGG) database (http://www.genome.jp/kegg) was applied to analyze the related pathways.

### 2.7 16S rRNA microbial community analysis

Microbial DNA was extracted from feces using a MoBio PowerSoil Kit. The concentration and purification of the final DNA were assessed with a NanoDrop 2000 spectrophotometer. The V3–V4 hypervariable region of the bacterial 16S rRNA gene was selected for amplification with primer pairs 338F (5′-ACT​CCT​ACG​GGA​GGC​AGC​AG-3′) and 806R (5′-GGACTACHVGGGTWTCTAAT-3′) using a PCR thermocycler system (GeneAmp 9,700, ABI, United States). PCR amplification cycling conditions were as follows: an initial denaturation at 95°C for 3 min, followed by 27 cycles of denaturation at 95°C for 30 s, annealing at 55°C for 30 s, extension at 72°Cfor 45 s, and single extension at 72°C for 10 min, and end at 4°C. All samples were amplified in triplicate. The PCR products were extracted from a 2% agarose gel, purified by AxyPrep DNA Gel Extraction Kit and quantified with a QuantiFluor™-Fluorometer (Promega, United States). The Illumina MiSeq PE300 platform (Illumina, San Diego, United States) was used for paired-end sequencing according to the standard protocols. The Fastp program (https://github.com/OpenGene/fastp, version 0.19.6) was used to normalize the quality of the original sequences, and Flash software was used (http://www.cbcb.umd.edu/software/flash, version 1.2.11) for splicing. The valid reads from all samples were clustered into operational taxonomic units (OTUs) at a 97% sequence similarity level using UPARSE 11**.** The taxonomy of each OTU representative sequence was analyzed with RDP Classifier version 2.13 against the 16S rRNA gene database using a confidence threshold of 0.7.

Bioinformatic analysis of the gut microbiota was conducted on the Majorbio Cloud platform (https://cloud.majorbio.com). Using the OTU information, rarefaction curves and alpha diversity indices including observed OTUs, the Coverage index and Simpson index were calculated with Mothur v1.30.2. The similarity among microbial communities in different samples was assessed through principal component analysis (PCA) and partial least squares discrimination analysis (PLS-DA) using Bray-Curtis dissimilarity with the Vegan v2.4.3 package. The linear discriminant analysis (LDA) effect size (LEfSe) (http://huttenhower.sph.harvard.edu/LEfSe) was performed to identify significantly abundant taxa (from phylum to genera) of bacteria among the different groups. The one-way analysis of variance analysis was conducted to screen species with significant differences at the genus level (*p* < 0.05).

### 2.8 Statistical analyses

The data are expressed as mean ± standard deviation (SD). The one-way analysis of variance (ANOVA) test was used to determine statistical significance between different groups using SPSS software (IBM, United States). A *p*-value <0.05 was considered statistically significant. Pearson correlation analysis was conducted by GraphPad Prism 7.0 with the Spearman index. The criteria for significance were a correlation coefficient |r|>0.70 and *p* < 0.05. Significant correlations between metabolites and gut microbe genera were obtained and displayed as heat maps.

## 3 Results

### 3.1 The component analysis of HP P-C

**FIGURE 1:**
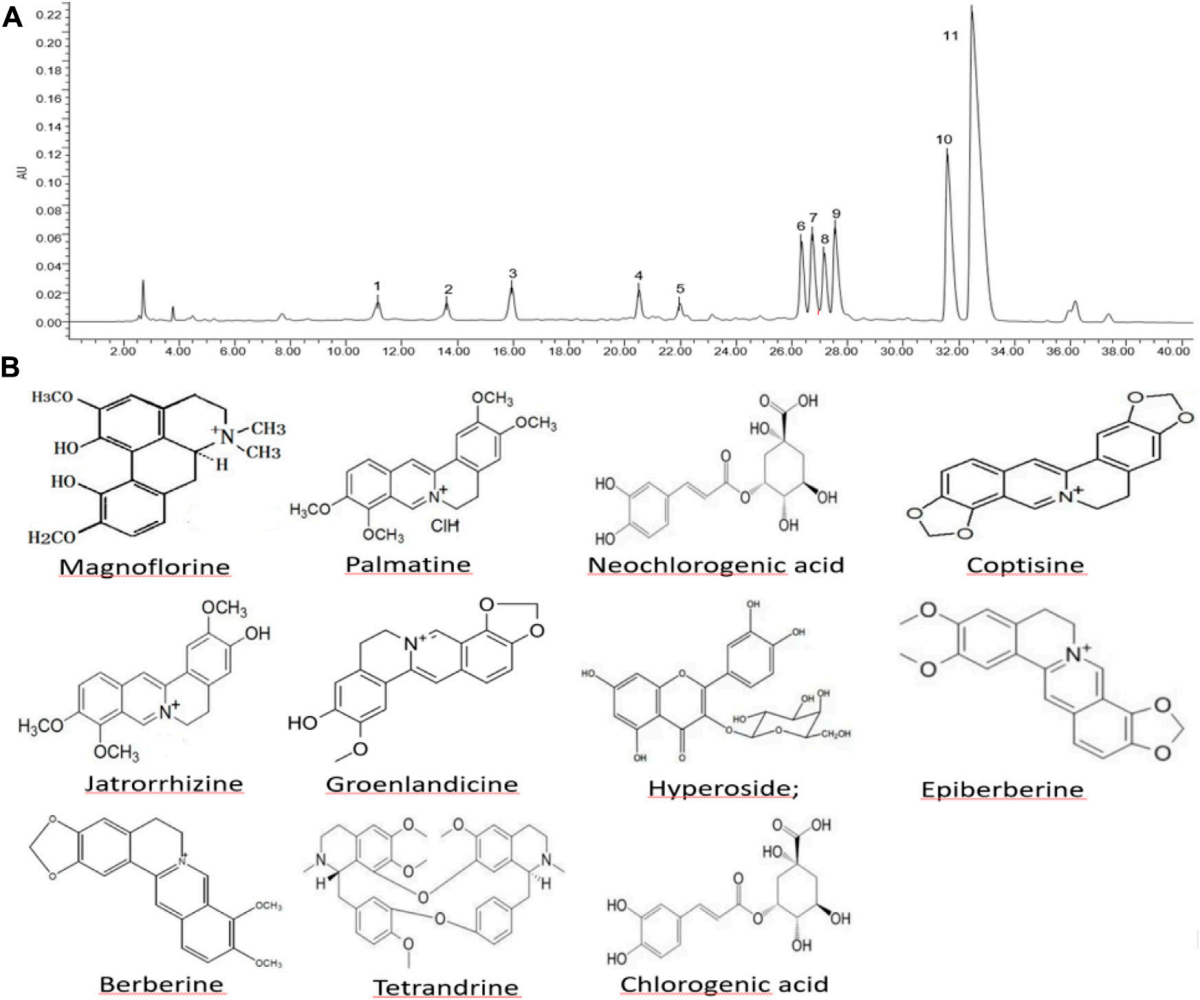
Composition and structure of major constituents in herbal pair *Polygonum hydropiper*-*Coptis chinensis*. **(A)** High-performance liquid chromatography of HP P-C. **(B)** Chemical structure of HP P–C: (1) neochlorogenic acid; (2) magnoflorine; (3) chlorogenic acid; (4) hyperoside; (5) groenlandicine; (6) tetrandrine; (7) jatrorrhizine; (8) coptisine; (9) epiberberine; (10) palmatine; (11) berberine.

The HP P-C was analyzed by HPLC and Eleven chemical components were identified, namely, neochlorogenic acid, magnoflorine, chlorogenic acid, hyperoside, groenlandicine, tetrandrine, jatrorrhizine, coptisine, epiberberine, palmatine and berberine, which are considered the main active ingredients (Figure 1).

### 3.2 HP P-C treatment mitigated WIRS-induced gastric mucosal injury in rats

**FIGURE 2:**
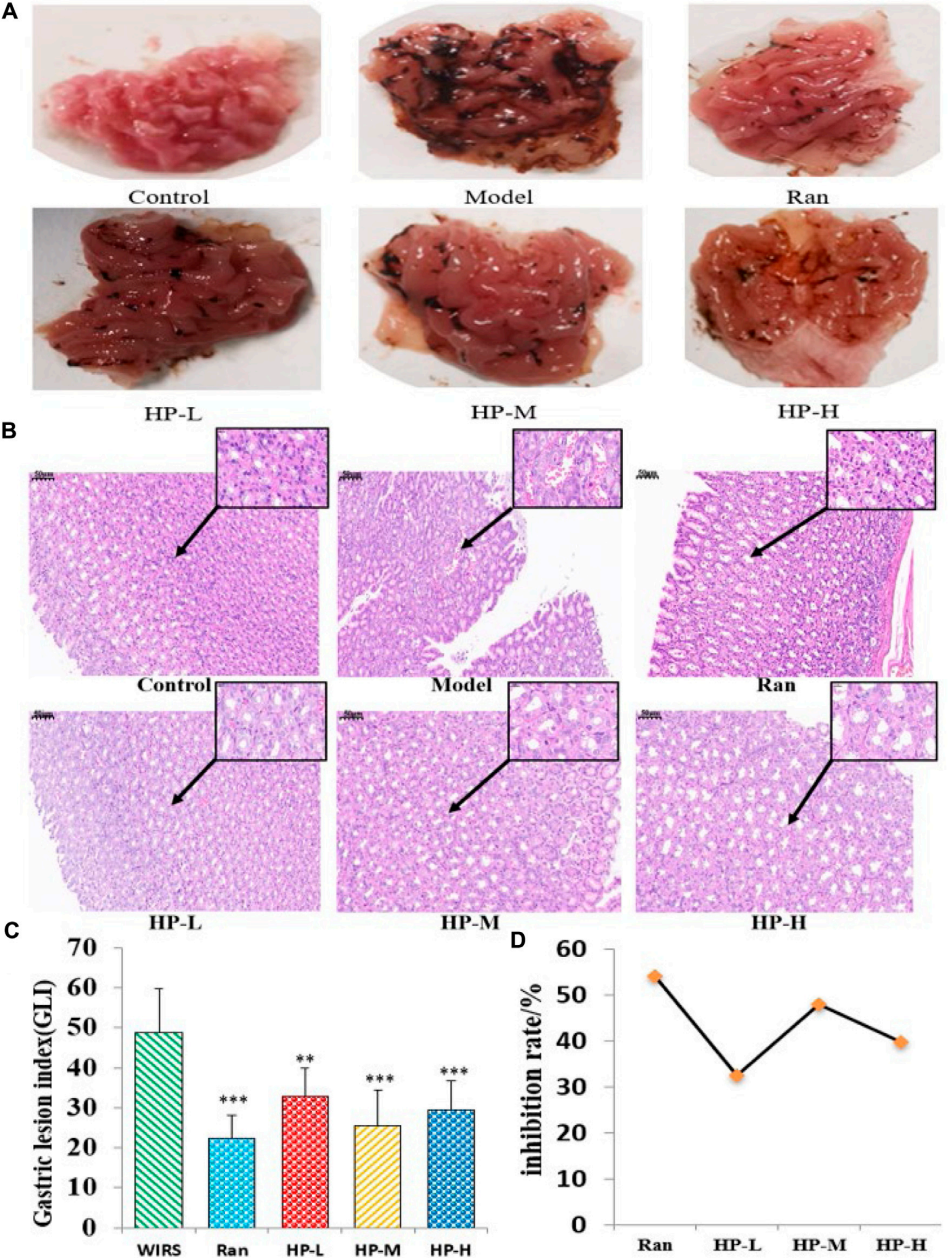
HP P-C reduced WIRS-induced gastric mucosal damage in rats. **(A)** Macroscopic appearance. **(B)** Histopathological analysis (HE staining, magnification: ×20, 100×). **(C and D)** Gastric lesion index and inhibition rate. All data are represented as means ± SD (n = 10). ^**^ *p* < 0.01 or ^***^ *p* < 0.001 vs WIRS group.

The effects of HP P-C on WIRS-induced gastric mucosal lesions were analyzed by observing macroscopic and histologic changes in the stomach. In Figure 2A, photographs of gastric tissue in each group are presented. The gastric mucosa appeared intact without impairment or bleeding in NC group. In contrast, the model group displayed apparent erosion and bleeding areas in the gastric antrum and body. The treatment group exhibited evidently lower levels of gastric mucosal bleeding compared to the WIRS group (Figure 2A). The pathological examination indicated that the WIRS-induced model exhibited remarkable injury to the gastric mucosal structure, including erosion, hemorrhage, edema, and mucosal cell shedding, while HP P-C treatment resulted in a nearly normal histological microstructure (Figure 2B). As depicted in Figure 2C, GLI was dramatically higher in WIRS group than in NC group, while HP P-C treatment effectively alleviated mucosal damage (Figures 2C,D).

### 3.3 HP P-C alleviated oxidative stress damage in WIRS rat

**FIGURE 3:**
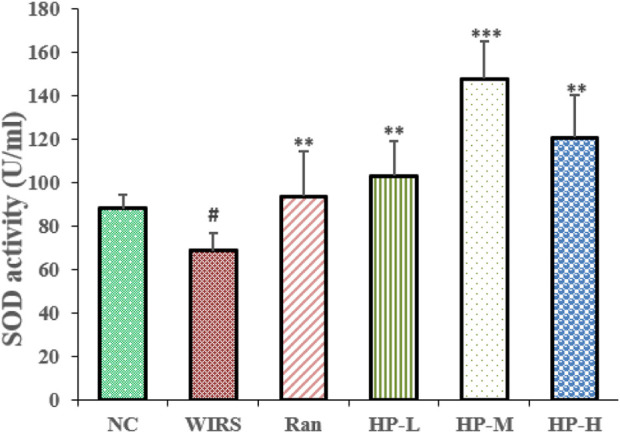
HP P-C alleviated WIRS-induced oxidative stress in rats. SOD activity and NO level in the serum samples obtained from the indicated groups. All data are represented as means ± SD (n = 10). ^#^ *p* < 0.05 vs NC group; * *p* < 0.05, ** *p* < 0.01 or *** *p* < 0.001 vs WIRS group.

Oxidative stress markers were used to evaluate the effects of HP P-C on WIRS-induced gastric injury. As shown in Figure 3A, the SOD activity in the model group was lower than that in NC group. Additionally, the SOD activity in the three HP P-C groups was significantly higher than that in the WIRS group, with the most notable increase observed in the medium and high dose groups (Figure 3). The results suggest that HP P-C has the potential to mitigate WIRS-induced gastric mucosal injury in rats.

### 3.4 Analysis of HP P-C modulating rat fecal metabolism

**FIGURE 4:**
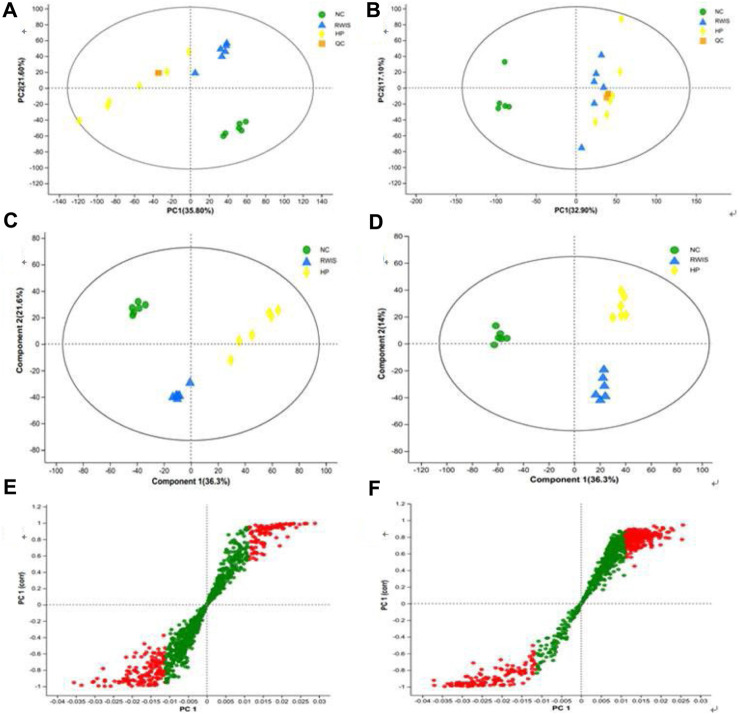
PCA **(A and B)** and PLS-DA **(C and D)** score plots of fecal metabolic profiles in NC, WIRS and HP groups in positive mode **(A and C)** and negative mode **(B and D)**. S-Plot of **(E)** NC vs WIRS and **(F)** HP vs WIRS in positive mode. Red dots are recognized as potential differential metabolites (VIP >1, *p* < 0.05).

The principal component analysis (PCA) and partial least squares discriminant analysis (PLS-DA) models were utilized to analyze the variations in rat fecal metabolites across three groups. First, PCA was employed to investigate the comprehensive profile and overall changes of fecal metabolites following HP P-C administration. The results illustrated that NC group could be clearly distinguished from WIRS group, indicating shift in the endogenous substances after the establishment of the WRIS-induced model. Furthermore, separations were observed between the model and treatment groups (Figures 4A,B), indicating that the metabolic profile of feces changed to varying degrees post the administration of HP P-C. PLS-DA was then employed to further determine the differences and changes in endogenous metabolites in feces between NC and WIRS groups and the HP P-C and model groups (Figures 4C,D). The plots displayed distinct separations between NC and WIRS groups, as well as the HP P-C and WIRS groups in both positive ion and negative ion modes (NC vs model: R^2^X = 0.652, R^2^Y = 0.998, Q^2^ = 0.984; model vs HP P-C: R^2^X = 626, R^2^Y = 0.987, Q2 = 0.947). The findings highlighted obvious differences in stress-induced fecal metabolites compared to NC group, while treatment with HP P-C reduced these differences by adjusting stress-induced metabolite alterations. A dot in S-plots represents a variable, and red dots located away from the center are recognized as potential differential metabolites (Figures 4E,F).

**FIGURE 5:**
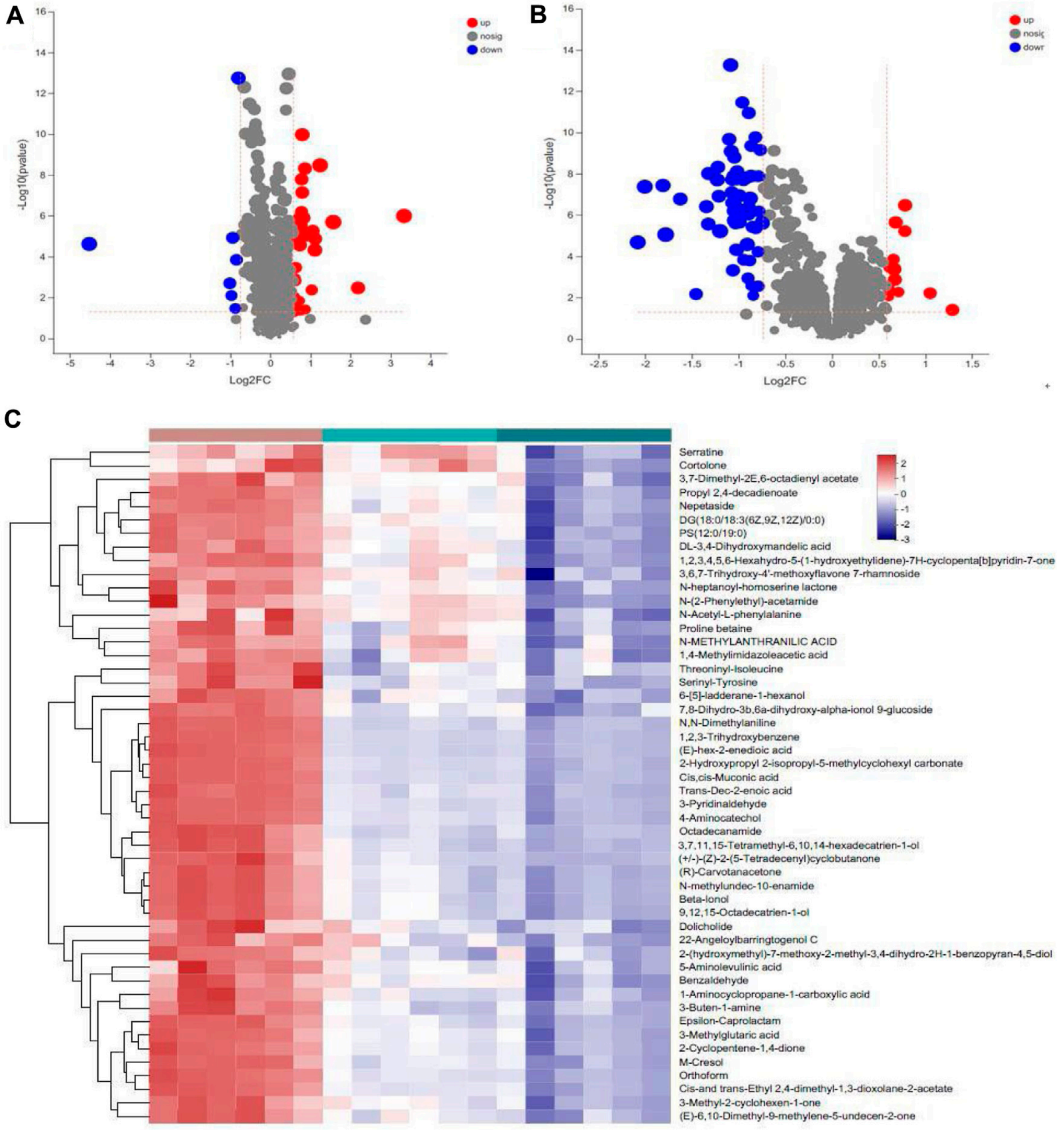
Volcano plot of metabolites identified using univariate statistical analysis that differed between NC and WIRS group **(A)**, WIRS and HP P-C group **(B)**. Significant changes in fecal metabolites are expressed as a heatmap **(C)** in the three groups.

Differential metabolites were screened based on the variable importance projection (VIP) parameters in the OPLS-DA model. T tests and univariate statistical analyses were carried out for the variables with VIP>1 and *p* < 0.05. Remarkably different metabolites were identified. A volcano plot was used to visualize the differential metabolites in the positive and negative ion modes. There were 30 metabolites with higher levels and seven metabolites with lower levels in WIRS group than in NC group (Figure 5A). There were 13 metabolites with higher levels and 59 metabolites with lower levels in the HP P-C group than in the model group (Figure 5B). Further analysis revealed 17 metabolites with significant changes in both sets differentially expressed metabolites (Table 1). To more visually represent the change in the levels of different metabolites in the samples obtained from each group, a heatmap analysis was conducted on the relative intensities of metabolite levels in NC, model and HP P-C rats (Figure 5C). The results from the identified differential metabolites were found to be representative of the differences observed.

**TABLE 1:** Identification and changes of metabolites in rat feces.

Metabolite	Formula	M/Z	VIP	FC(NC/WIRS)	*P*	VIP	FC(WIRS/HP)	*P*
L-Arginine	C6H14N4O2	175.1182	1.17	1.11↑	*	0.59	1.04↑	
L-Glutamine	C5H10N2O3	145.0607	1.14	1.11↑	**	0.77	0.94↓	
5-Hydroxyindoleacetaldehyde	C10H9NO2	208.0965	1.36	0.87↓	*	1.35	1.23↑	**
4-(2-Amino-3-hydroxyphenyl)-2,4-dioxobutanoic acid	C10H9NO5	222.0403	1.61	1.30↑	***	2.88	0.67↓	***
5-Hydroxyindoleacetic acid	C10H9NO3	190.0496	1.01	1.10↑	***	1.59	0.84↓	*
4-(2-Aminophenyl)-2,4-dioxobutanoic acid	C10H9NO4	206.0442	1.61	1.30↑		2.04	0.79↓	**
Indole	C8H7N	118.0653	1.58	0.85↓		0.72	1.05↑	*
Tyramine	C8H11NO	273.1615	3.06	0.09↓	***	1.59	1.24↑	*
Gentisic acid	C7H6O4	153.0181	1.27	1.26↑	**	1.42	0.80↓	
L-Tyrosine	C9H11NO3	182.0810	1.54	0.85↓	***	1.17	1.14↑	**
L-Dopa	C9H11NO4	230.1021	2.09	0.68↓	***	1.42	1.29↑	**
Dopaquinone	C9H9NO4	176.0340	1.00	1.08↑		2.59	0.72↓	***
21-Deoxycortisol	C21H30O4	347.2207	0.63	0.94↓		1.15	1.14↑	**
Dihydrocortisol	C21H32O5	363.2171	0.63	0.95↓		1.32	1.07↑	**
6-Hydroxyhexanoic acid	C6H12O3	113.0596	1.19	1.27↑	***	1.85	0.74↓	***
N-Methyl-L-glutamate	C6H11NO4	340.1743	1.38	0.86↓	***	1.15	1.19↑	*
L-Tyrosine methyl ester	C10H13NO3	196.0967	1.52	0.86↓	***	1.09	0.11↓	**

FC: fold change. * *p* < 0.05, ** *p* < 0.01, ***p < 0.001 vs WIRS group.

**FIGURE 6:**
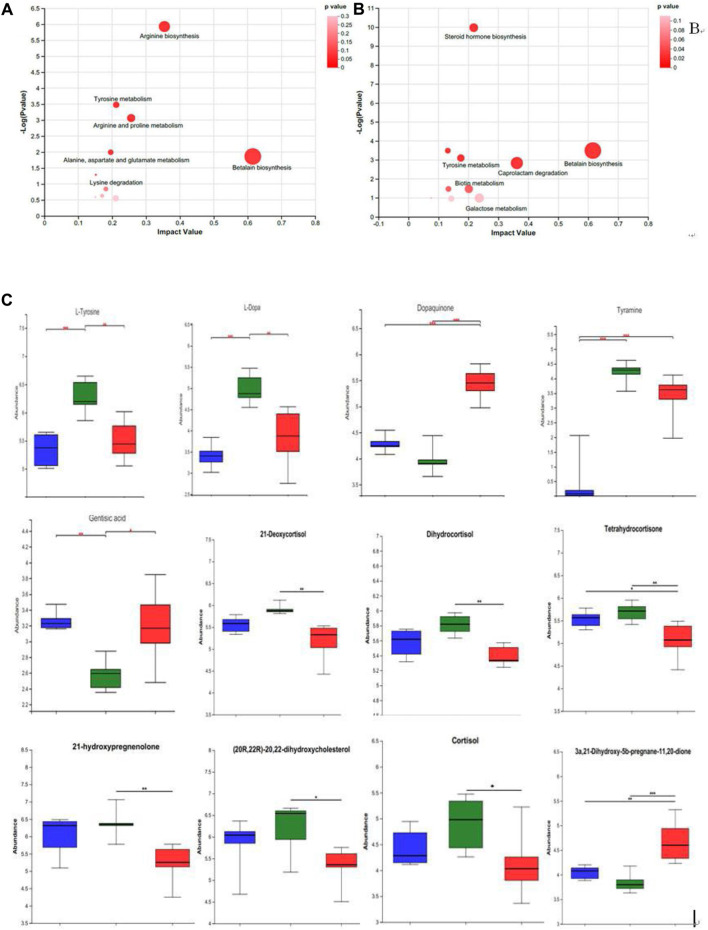
Summary of pathway analysis using MetPA in NC and WRIS group **(A)**, WRIS group and HP group **(B)**. Relative intensities of potential biomarkers in different groups **(C)**. (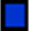)NC group; (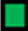)WRIS group; (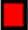)HP group. * *p* < 0.05, ** *p* < 0.01, *** *p* < 0.001.

The prominently differential metabolites were selected as potential biomarkers, and the most relevant pathways influenced by WRIS model and HP P-C treatment were investigated. As shown in Figures 6A,B, six main metabolic pathways were influenced by WRIS model, including betalain biosynthesis; arginine biosynthesis; arginine and proline metabolism; tyrosine metabolism; alanine, aspartate and glutamate metabolism; and lysine degradation, while HP P-C treatment affected six important metabolic pathways, such as betalain biosynthesis, steroid hormone biosynthesis, tyrosine metabolism, biotin metabolism, caprolactam degradation and galactose metabolism. Interestingly, there were two pathways in common among NC, WIRS and HP P-C groups, namely, tyrosine metabolism and betalain biosynthesis. The metabolites with significant differences in these pathways are tyramine and gentisic acid in tyrosine metabolism, as well as L-tyrosine, L-dopa, and dopamine in betaine biosynthesis (Figures 6A,B). Furthermore, HP P-C treatment influenced the biosynthesis of steroid hormones, affecting metabolites like (21-deoxycortisol, dihydrocortisol, tetrahydrocortisone, 21-hydroxypregnenolone, (20R,22R)-20,22-dihydroxycholesterol, cortisol, and 3a, 21-dihydroxy-5b-pregnane-11,20-dione (Figure 6C).

### 3.5 HP P-C increased the richness and diversity of the gut microbiota community

**FIGURE 7:**
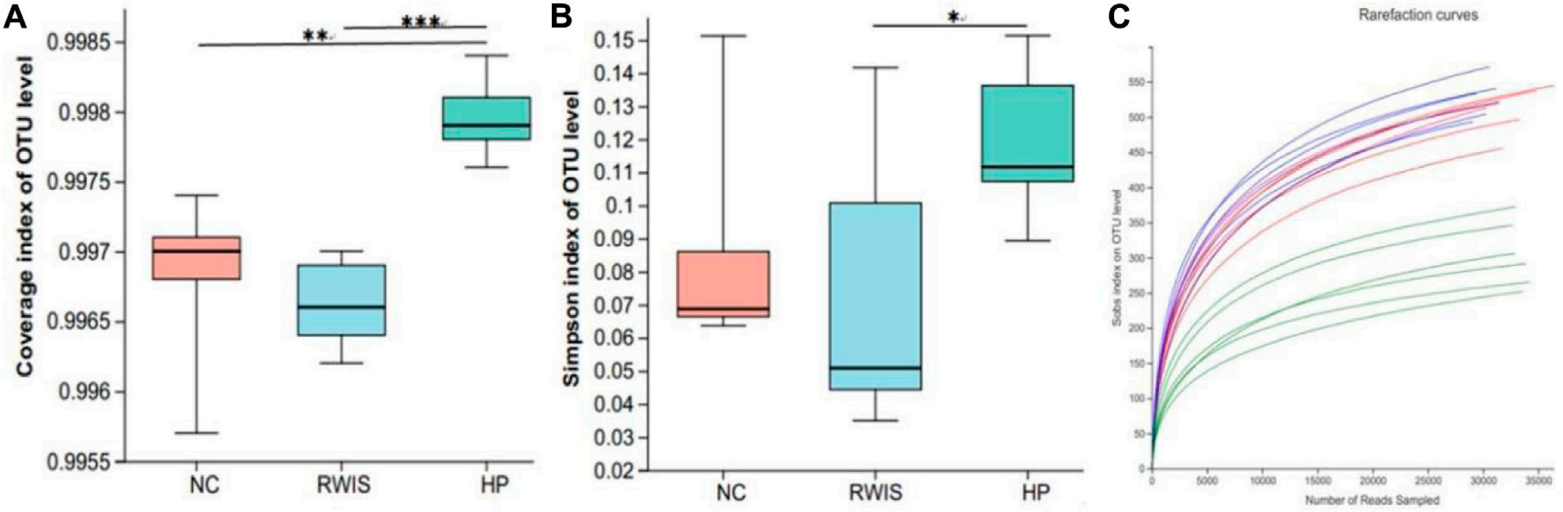
Impact of HP P-C on alpha diversity of the gut microbiota. Coverage index **(A)**. Simpson index **(B)**. Rarefaction curve analysis **(C)**. * *p* < 0.05, ** *p* < 0.01, *** *p* < 0.001.

Alpha diversity analysis was ed using the results of 16S rRNA sequencing based on a 97% similarity level. The Simpson index was used to analyze species diversity. Generally, result of the alpha diversity index illustrated that the microbial flora diversity in WIRS group was not significant different from that in NC group, indicating that the stress stimulus manifested little effect on the diversity of the microbial flora (Figure 7A), while HP P-C treatment notably increased the diversity of the microbial populations (Figure 7B). Rarefaction curves tended toward flatness with the increase in the number of sampled, exhibiting that the sequencing depth was sufficient to capture the information on most microbial species (Figure 7C).

### 3.6 HP P-C altered the structure and composition of the gut microbiota

**FIGURE 8:**
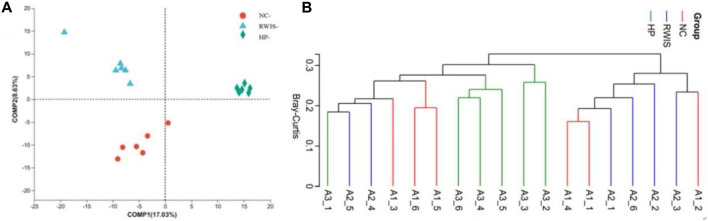
PLS-DA **(A)** and hierarchical clustering **(B)** analyses between the bacterial communities in the three groups.

The composition of the intestinal microflora among the three groups was further analyzed to investigate the influence of stress and HP P-C on the structure of the gut microbiota using PLS-DA. As shown by the PLS-DA plots in Figure 8A, the model group showed complete departure from NC group and tended toward different quadrants (Figure 8A). This significant difference indicate that stress caused notable alterations in the gut flora structure in rats, while the HP P-C treatment group showed a complete divergence from the model group, indicating a pronounced influence HP P-C on the microorganism structure. The Bray‒Curtis revealed that animals receiving HP P-C clustered in the middle (Figure 8B), while those subjected to stress clustered on the right side, and control rats clustered on the left side. The results suggest that HP P-C has an obvious bearing on the bacterial community structure.

**FIGURE 9:**
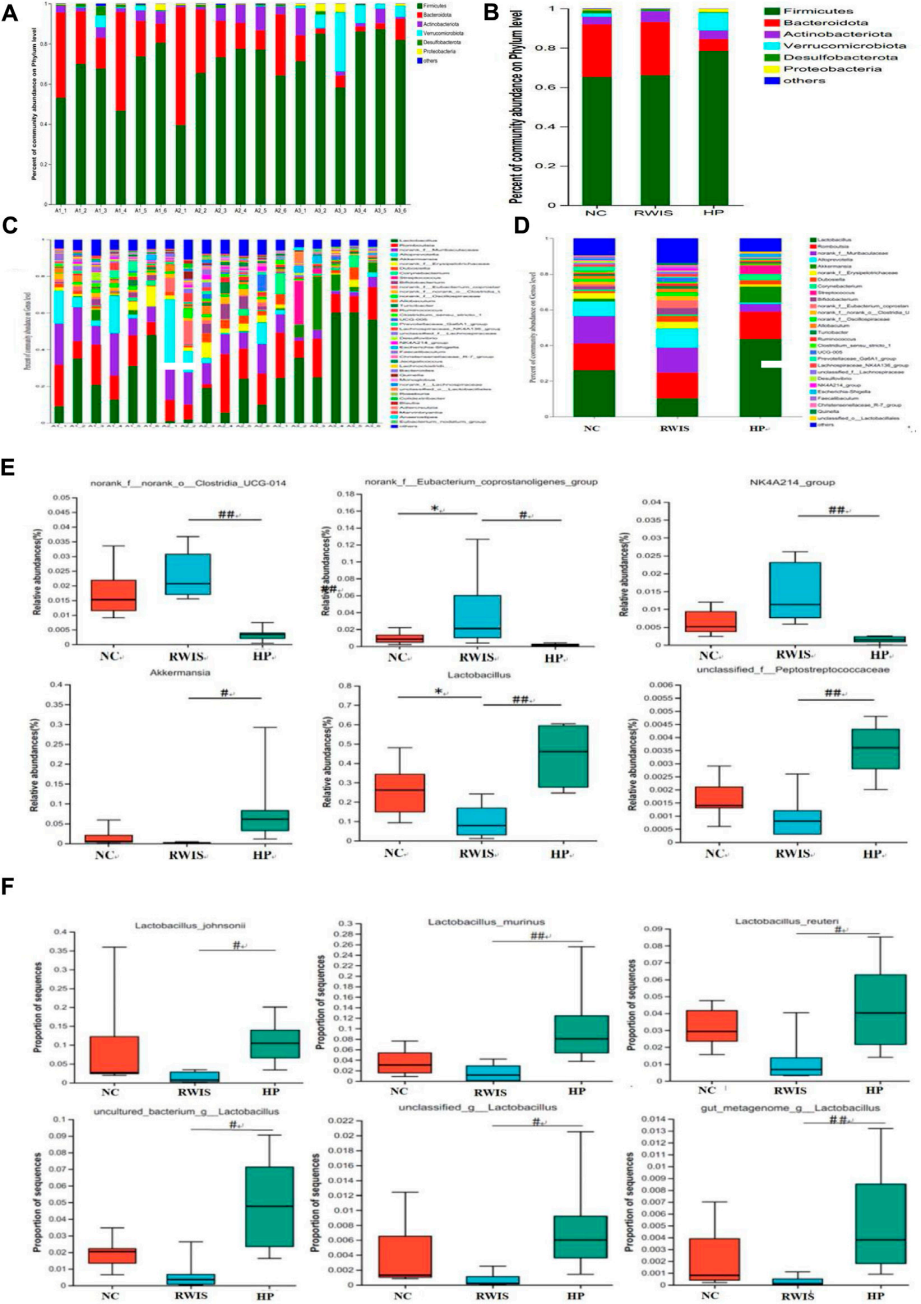
The impact of HP P-C on the gut microbial profile at the phylum **(A and B)** and genus **(C and D)** levels. Typical genera with significant changes in abundance **(E)**. *Lactobacillus* with high relative abundance **(F)**. All data are represented as means ± SD (n = 6). * *p* < 0.05 vs NC; ^#^ *p* < 0.05 and ^##^ *p* < 0.01 vs WIRS.

The microbial profiles were analyzed at the phylum and genus levels. Generally, HP P-C treatment altered the composition at the microbial genus level more obviously than at the phylum level. At the phylum level (Figures 9A,B), the composition of dominant bacteria in each group was basically similar, with Firmicutes and Bacteroidetes being two dominant phyla. The abundance of *Verrucomicrobiota* was significantly lower in WIRS group than in NC group, while after HP P-C treatment, the abundance of *Verrucomicrobiota* reversed, and there was an observably higher abundance of *Firmicutes* in the HP P-C group compared to WIRS group. At the genus level (Figures 9C,D), the top five genera with high relative abundances among the three groups were *Lactobacillus, Romboutsia, norank_f_Muribaculaceae*, *Alloprevotella* and *Akkermansia* (AKK). The abundance of *Lactobacillus* and *Akkermania* was prominently lower in WIRS group than in NC group, while the abundance of *Eubacterium_coprostanoligenes* and *NK4A214* was higher in WIRS group. In contrast, the abundance of *Lactobacillus, Akkermannia* and Peptostreptococcaceae in the HP P-C group was markedly higher, while that of *Clostridia_UCG-014, Eubacterium_coprostanoligenes* and *NK4A214* was significantly lower (Figure 9E). These distinct responses of individual genera to HP P-C showed that HP P-C has a clear modulatory effect on the gut microbiota, highlighting *Lactobacillus* and *Akkermannia* may be the key genera for HP P-C treatment of stress-induced gastric ulcers.

In addition, the study also delved into the changes in *Lactobacillus* composition. HP P-C remarkablly affect *Lactobacillus* and *Akkermansia*, resulting in a sharp increase in abundance of *Lactobacillus* in rats treated with HP P-C. The identified *Lactobacillus* species comprise *Lactobacillus_murinus, Lactobacillus johnsonii, Lactobacillus reuteri, gut_metagenome_g_Lactobacillus, uncultured_bacterium_g_Lactobacillus* and unclassified_*g_Lactobacillu*s (Figure 9F). These findings suggest that HP P-C prominently inverted the microbial dysbiosis in stress-induced rats, restoring them to control conditions. This discovery manifested that the changes in HP P-C-regulated gut microbial flora may contribute to the enhancement of gastric function.

**FIGURE 10:**
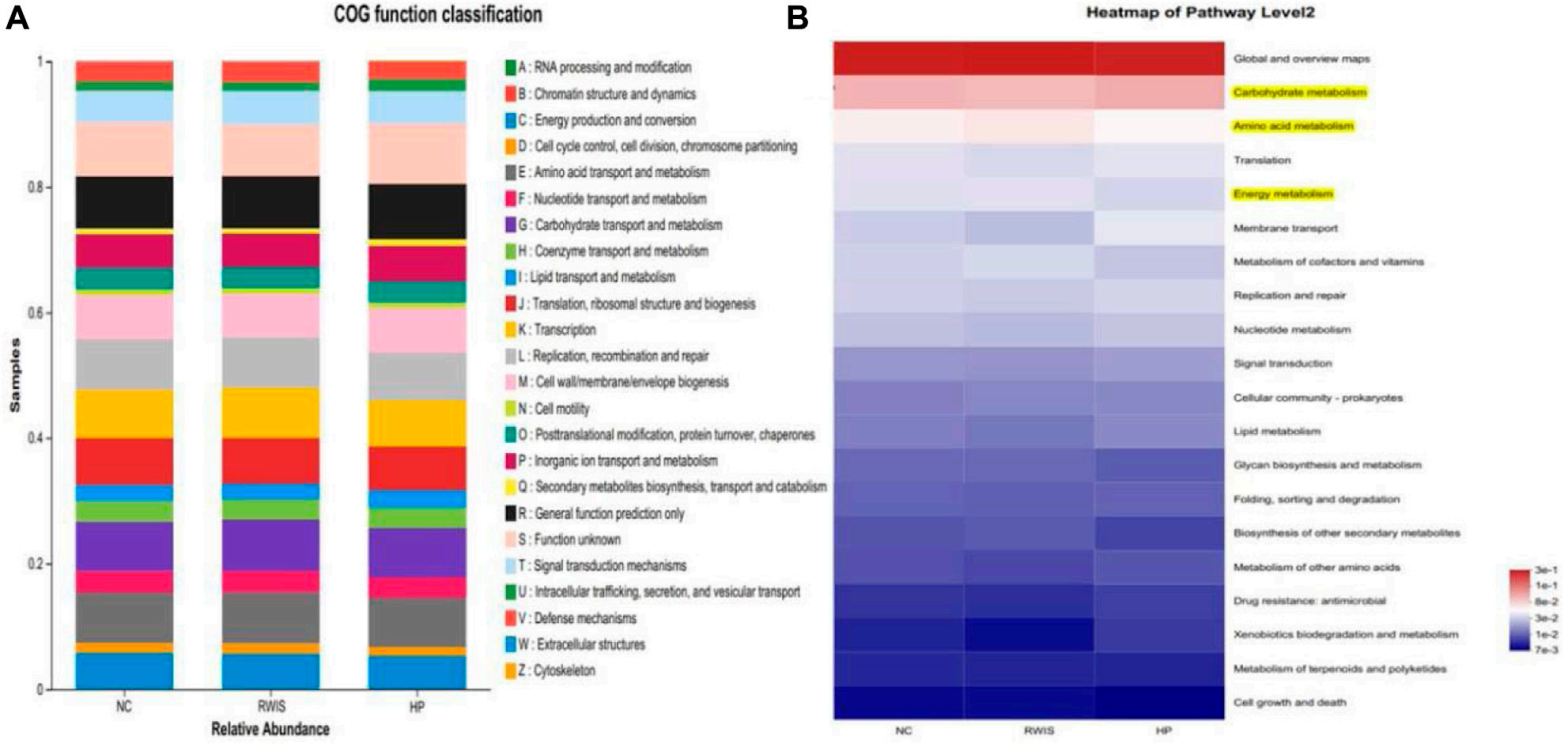
Analysis based on COG and KEGG database used to predict microbial metabolic function **(A and B)**.

Microbiome metabolic function was predicted based on Clusters of Orthologous Groups (COG) and Kyoto Encyclopedia of Genes and Genomes (KEGG) analysis at the genus level among the three groups. To examine the regulatory activity of HP P-C on the gut microbial community of WRIS rats, we compared the differences in microbial metabolic functions among the three groups. Differential microbes were primarily related to amino acid transport and metabolism, carbohydrate transport and metabolism, energy metabolism and cell wall/membrane/envelope biogenesis (Figure 10A). The pathways annotated with yellow in Figure 10B designated potential pathways interrelated with alterations in the gut microorganisms of rats treated with HP P-C (Figure 10B).

### 3.7 Correlation analysis of gut microbes and fecal metabolites

**FIGURE 11:**
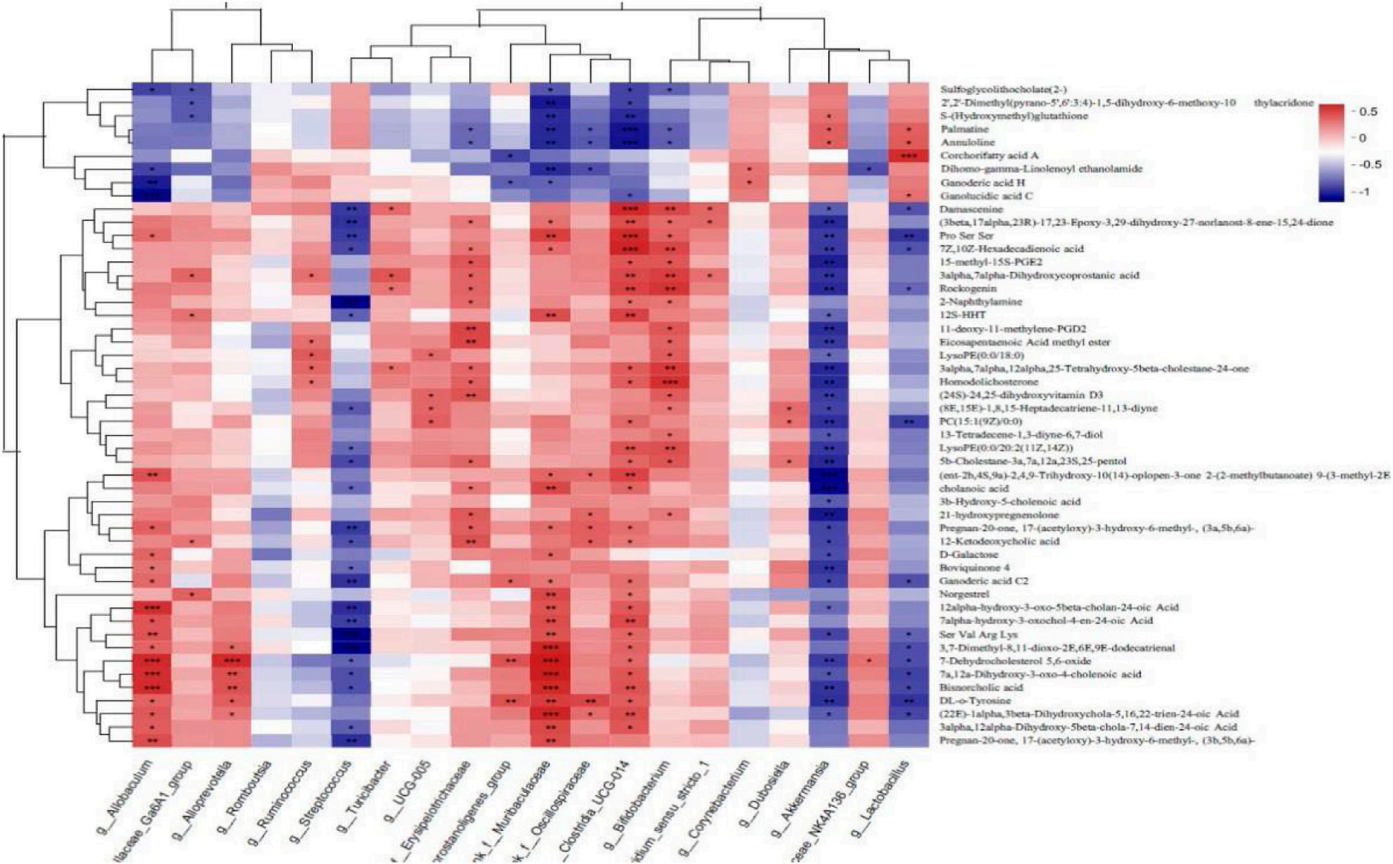
Correlation analysis of microbiota and metabolites. *p < 0.05, **p < 0.01, ***p < 0.001.

Spearman correlation analysis was carried out to analyze the correlation in the levels of the top 50 differentially abundant metabolites and the top 20 differentially abundant bacterial populations between the WIRS and HP P-C groups. As illustrated in Figure 11, at the genus level, the alteration in the abundance of *Akkermansia* and *Lactobacillus* were negatively bound up with changes in tyrosine, 21-hydroxypregnenolone, D-galactose and cholenoic acid levels, while positively correlated with S-glutathione, corchorifatty acid, ganolucidic acid and palmatine levels (Figure 11). This suggests that HP P-C may reduce tyrosine and 21-hydroxypregnenolone levels while increasing S-glutathione concentration by modulating the abundance of *Akkermansia* and *Lactobacillus*, thereby enhancing blood supply and antioxidant capacity to fight against stress-induced gastric mucosal injury.

## 4 Discussion

Stress is among the important factors involved in ulcer formation, leading to acute mucosal bleeding and high mortality (Krag et al., 2015). Moreover, it is well known that excessive physical or psychological stress can trigger gastric lesions in both humans and animals (Levenstein et al., 2015). The water-immersion restraint stress (WIRS) model is extensively regarded as a method to study stress-related gastric damage and may imitate the clinical acute gastric injury induced by serious infections, surgery, or shock. The main pathological changes observed in WIRS-induced gastric damage are hyperemia, hemorrhage and erosions in the mucosa and submucosa of the stomach. In this study, the WIRS-induced rat model exhibited pathological changes consistent with these seen in stress-related gastric ulcer disease. The impact of HP P-C on gastric injury was investigated *in vivo*, revealing a clear protective effect on gastric injury induced by WIRS. This was evidenced by an obvious reduction in the GLI score and an improvement in gastric mucosa damage. The histological examination revealed that 6 hours of WIRS loading in model rats resulted in significant hemorrhagic injury in the glandular stomach, after oral administration of HP P-C, gastric mucosal congestion and bleeding were significantly reduced.

It has been demonstrated that exposure to stress conditions can provoke multiple pathways, resulting in an overproduction of free radicals as a result of an imbalance between the body’s oxidative and antioxidant systems. Excessive free radicals can cause membrane lipid peroxidation, DNA damage, and cell death, and contribute to the occurrence of pathological conditions, including exacerbating mucosal injury (Wu et al., 2023). SOD is the key enzyme of the antioxidant defense system by converting superoxide radicals into hydrogen peroxide (H_2_O_2_). Previous research has demonstrated that reactive oxygen species (ROS) are participated in the pathogenesis of ethanol-induced gastric ulcers and scavenging the reactive oxygen species can ameliorate gastric injury (Tahir et al., 2022). The current study demonstrated that pretreatment with HP P-C obviously raised the SOD activity in stressed rats, contributing to scavenging of free radical and reduction in oxidative lesions in the gastric mucosa of rats subjected to stress. Thus, HP P-C exerted a gastroprotective effect, probably due to its enhanced antioxidant capacity resulting from the increased SOD activity.

TCM can exert pharmacological effects by regulating the gut microbial community and fecal metabolites through its chemical constituents (Han et al., 2024). Various active ingredients in TCM have demonstrated the ability to influence gut microbiota and fecal metabolites (Gan et al., 2024). HPLC analysis of HP P-C extracts aimed to evaluate whether its regulation of gut bacteria and metabolites was linked to specific chemical compounds. Berberine, palmatine, coptisine, jatrorrhizine and hyperoside are the main active ingredient of HP P-C. The regulatory action of HP P-C on metabolites and the gut microbial flora was closely interrelated to its active constituents. It has been well known that berberine can regulate gut microorganisms (Zhang et al., 2024).

The dynamic balance of intestinal microorganisms is intricately related to human health (Fan and Pendersen, 2021). The gut microbiota plays a vital role in maintaining homeostasis and health in humans (Fassarella et al., 2021). Gut microbiota dysbiosis and metabolic phenotype alteration are closely connected to the development of various diseases (Lavelle and Sokol, 2020), such as gastric ulcer (Gus) and inflammatory bowel disease (IBD). Through interacting with the host, the gut microbiota acts on target organs to modulate the homeostasis of the host and the development of diseases. The regulation of gut flora dysbiosis by medicated food and TCM has been shown to have beneficial effects in sustaining the integrity of intestinal mucosa and alleviating gastric injury (Feng et al., 2019). It has been reported that disruptions in intestinal microflora have been implicated in the gastric mucosal damage caused by WIRS (Wang et al., 2020). In addition, evidence also has been found that TCM, containing myriad compounds, unavoidably interact with the gut microbiota post-oral administration to restore the homeostasis of the gut microbiota and alleviate pathological damage and clinical symptoms (Yue et al., 2019; Huang et al., 2023) by modulating the structure and metabolism of gut microbiota. The intestinal microbiota can enhance its efficacy (Xu et al., 2023), weaken toxicity, and avoid adverse drug reactions by metabolizing or altering the active ingredients of TCM. Based on this knowledge, we have reason to believe that HP P-C directly or indirectly induces beneficial changes in the gut flora, thereby reducing gastrointestinal damage. The results showed that oral administration of HP P-C had remarkable influence on the structure of the gut microbiota. Alpha diversity analysis proved that HP P-C notably raised the abundance and diversity of the gut microbiota. The richness and diversity were lower in model rats than in NC rats, and HP P-C treatment elevated the gut microbiota diversity. Beta diversity analysis illustrated that HP P-C has analogous influence on gut microbiota composition, leading to obvious alteration in the overall structure of the gut microbiota in HP P-C-treated rats compared to NC group. There were clear taxonomical differences in *Verrucomicrobiota* at the phylum level. The abundance of *Verrucomicrobiota* decreased in stress rats, while that in animals treated with HP P-C showed completely opposite changes. Furthermore, oral administration of HP P-C increased the abundance of Firmicutes, with stress having little influence on this species. At the genus level, rats with HP P-C treatment had higher abundance of *Lactobacillus* and *Akkermania*, while the stressed rats had lower abundance of these beneficial bacteria. Gastrointestinal diseases have direct relationship with changes in intestinal microorganisms, with different bacteria exerting distinct impacts on host health. *Lactobacillus* alleviates inflammation, prevents pathogen invasion and *Clostridium difficile* infection (Nowak et al., 2019), activates phagocytosis in macrophages, and boost immunity. The pharmacological effects of *Akkerman*ia involve relieving intestinal inflammation, delaying aging, lowering blood lipid and glucose levels, improving immunity, regulating nervous system function, and exerting anti-inflammatory and anticancer effects (Zhai et al., 2019). Its mechanism of action involves improving the thickness of intestinal mucus, maintaining the integrity of the intestinal barrier, and preventing the invasion of foreign pathogen or toxic substance. TCM has important effects on various gastrointestinal diseases by targeting the intestinal flora. *Lactobacillus* and AKK bacteria may be key bacteria of TCM interventions, with a notable rise in AKK bacterial levels after treatment with TCM serving as compelling evidence of its efficacy (Zhou, 2017). These findings indicated that changes in the HP P-C-regulated gut microbiota were correlate to the enhancements in gastric function.

Targeted metabolomics was used to assess the impact of TCM on fecal metabolites in stressed rats. UPLC-Q-TOF-MS/MS-based metabolomics is a powerful method for identifying potent metabolites. Seventeen endogenous metabolites were identified, and three main metabolic pathways (tyrosine metabolism, betalain biosynthesis and steroid hormone biosynthesis) were revealed.

Under stress, the sympathetic adrenal medullary system of rats is activated, leading to the release of high levels of catecholamines (Kopin et al., 1988). This causes vasoconstriction and a reduction in gastric mucosal blood flow (GMBF), aggravating gastric mucosal damage. Tyrosine serves as the precursor for catecholamines such as dopamine, epinephrine and norepinephrine. Through the catalysis of tyrosinase, tyrosine is converted into dopamine, which then further transforms into adrenaline and noradrenaline by specific enzymes enzyme. Alternatively, tyrosine can be converted into melanin or gentisc acid through other pathways. Our study revealed that following HP P-C administration, L-tyrosine and L-dopa levels decreased significantly, while gentisic acid and dopaquinone levels increased notably. Gentisic acid exhibits antipyretic and analgesic properties, while dopaquinone plays a role in an intermediate in the conversion of tyrosine into melanin. The results of this experiment suggest that HP P-C influences tyrosine metabolism by either reducing its synthesis and secretion or promoting its transformation into melanin or gentisic acid. Consequently, HP P-C can alleviate the vasoconstriction of the gastric mucosa and improve the microcirculation of the gastric mucosa.

Stress can enhance the activity of the hypothalamic-pituitary-adrenal (HPA) axis (Zhang et al., 2012), leading to an increase in the release of glucocorticoids (GCs). While appropriate level of glucocorticoids can alleviate the stress response and enhance the body’s ability to cope with harsh environments (Kramer et al., 2019), however, prolonged exposure to stress can result in excessive levels of GCs. Elevated GC levels can lead to organ dysfunction due to increased energy consumption. Furthermore, high GC levels can suppress the immune system, promote gastric acid secretion, and exacerbate damage to the gastric mucosa (Filaretova et al., 2009). The findings of this study revealed that HP P-C can modulate the metabolism of steroid hormones in stressed rats. Specifically, the levels of 21-deoxycortisol, 21-hydroxypregnenolone, (20R, 22R)-20, 22-dihydroxycholesterol, cortisol, dihydrocortisol and tetrahydrocortisone obviously decreased, while the level of 3a, 21-dihydroxy-5b-pregnane-11,20-dione notably increased in rats given HP P-C by gavage. Notably, the 21-deoxycortisol, 21-hydroxypregnenolone and (20R, 22R)-20, 22-dihydroxycholesterol serve as precursors to adrenocortical hormones, whereas cortisol functions as a kind of glucocorticoid. Dihydrocortisol, tetrahydrocortisone and 3a, 21-dihydroxy-5b-pregnane-11, 20-dione represent metabolites of cortisol or corticosterone. These results suggest that HP P-C can regulate of glucocorticoids production, attenuate their impact on gastric acid secretion, and reduce damage to the gastric mucosa.

The limitations of this study are that the screened differential intestinal flora and metabolites that were have not yet been validated. Given the current research results, further exploration is required to fully understand the relationship between the protective effect of HP P-C on gastric damage and the differential gut microbiota and metabolites in the future.

## 5 Conclusion

The results obtained in this work showed that HP P-C exhibits a protective effect on stress-induced gastric mucosal damage in rats, which may be related to regulating oxidative stress, improving mucosal blood circulation and protecting the gastric mucosa. In addition, HP P-C regulated the gut microbiota structure and metabolic phenotype of the host during the treatment of stressed rats. The effects of HP P-C on stress-induced gastric injury may be involved with a series of metabolite changes, including changes in tyrosine metabolism, betalain biosynthesis and steroid hormone biosynthesis, although further investigation is needed. The relationship between gut microbiota and metabolites may provide a novel perspective to explore stress-related gastric lesions. Overall, these results suggested that HP P-C can inhibit gastric damage induced by restraint water-immersion stress while regulating disorders of the gut microbiota and metabolites and shows potential for treatment of diseases related to gastric mucosal injury.

## Data Availability

The original contributions presented in the study are publicly available. This data was deposited into the NCBI Sequence Read Archive (SRA) database (accession number: SRP524930).
